# Bioinformatics and machine learning approaches reveal key genes and underlying molecular mechanisms of atherosclerosis: A review

**DOI:** 10.1097/MD.0000000000038744

**Published:** 2024-08-02

**Authors:** Xiaoxue Su, Meng Zhang, Guinan Yang, Xuebin Cui, Xiaoqing Yuan, Liunianbo Du, Yuanmin Pei

**Affiliations:** a Vascular Surgery Department of Weifang Yidu Central Hospital, Weifang, Shandong, China; b Department of Urology, People’s Hospital of Qingdao West Coast New Area, Qingdao, Shandong, China; c Kingmed Diagnostics, Guangzhou, Guangdong, China; d Dalian Medical University, Dalian, China.

**Keywords:** atherosclerosis, CMAP, GO, GSEA, GSVA, immune infiltration, KEGG, machine learning, scRNA-seq

## Abstract

Atherosclerosis (AS) causes thickening and hardening of the arterial wall due to accumulation of extracellular matrix, cholesterol, and cells. In this study, we used comprehensive bioinformatics tools and machine learning approaches to explore key genes and molecular network mechanisms underlying AS in multiple data sets. Next, we analyzed the correlation between AS and immune fine cell infiltration, and finally performed drug prediction for the disease. We downloaded GSE20129 and GSE90074 datasets from the Gene expression Omnibus database, then employed the Cell-type Identification By Estimating Relative Subsets Of RNA Transcripts algorithm to analyze 22 immune cells. To enrich for functional characteristics, the black module correlated most strongly with T cells was screened with weighted gene co-expression networks analysis. Functional enrichment analysis revealed that the genes were mainly enriched in cell adhesion and T-cell-related pathways, as well as NF-κ B signaling. We employed the Lasso regression and random forest algorithms to screen out 5 intersection genes (CCDC106, RASL11A, RIC3, SPON1, and TMEM144). Pathway analysis in gene set variation analysis and gene set enrichment analysis revealed that the key genes were mainly enriched in inflammation, and immunity, among others. The selected key genes were analyzed by single-cell RNA sequencing technology. We also analyzed differential expression between these 5 key genes and those involved in iron death. We found that ferroptosis genes ACSL4, CBS, FTH1 and TFRC were differentially expressed between AS and the control groups, RIC3 and FTH1 were significantly negatively correlated, whereas SPON1 and VDAC3 were significantly positively correlated. Finally, we used the Connectivity Map database for drug prediction. These results provide new insights into AS genetic regulation.

## 1. Introduction

Among the global leading causes of death is cardiovascular disease caused by atherosclerosis (AS).^[1,2]^ In AS, arterial walls are thickened and hardened due to the accumulation of cells, cholesterol, and the extracellular matrix.^[3,4]^ The common characteristics of all kinds of arteriosclerosis are thickening and hardening of arterial wall, loss of elasticity and narrowing of lumen. High risk factors include hyperlipidemia, hypertension, smoking, diabetes, and obesity.^[5,6]^ According to the “endothelium damage response theory,” several major risk factors for cardiovascular disease eventually cause damage to the intima of arteries,^[7]^ while AS results from inflammation and fibroproliferative reactions in the artery following injury to the endothelium and intima.^[8,9]^ It is not difficult to diagnose this disease when it develops to a certain extent, especially when there are obvious organ lesions. However, early diagnosis remains a challenge. Studies have shown that half the risk of AS is determined by genes.^[10,11]^ Therefore, identification of additional key genes and the molecular mechanisms underlying its development is imperative to effective management of the disease.

Numerous studies have shown that immune cells infiltrate the vascular wall and contribute to AS progression of.^[12–15]^ Therefore, elucidating the role of immune cell infiltration in AS development is imperative to understanding its molecular pathogenesis from an immunity perspective. Several studies have employed sequencing single cells of atherosclerotic arteries to identify immune cell subsets.^[12,16,17]^ Although atherosclerotic lesions are associated with a dysfunctional immune system, the specific characteristics of these cells and effective biomarkers for AS remain unclear. Bioinformatics analysis and data mining have been applied in many fields, such as the use of high-throughput screening technology in gene sequencing, and gene microarray expression analysis to clarify the molecular mechanism and diagnostic markers of diseases. These tools have also been used to detect single nucleotide polymorphisms,^[18–21]^ thus effectively promoting medical research.

In this study, we employed bioinformatics and machine learning tools to explore key genes and molecular mechanisms underlying AS development in multiple data sets. Next, we examined their interactions with different immune cell infiltration states. We obtained 2 gene expression datasets, GSE20129 and GSE90074, from the Gene Expression Omnibus (GEO) database, an analyzed a total of 22 immune cells using the Cell-type Identification By Estimating Relative Subsets Of RNA Transcripts (CIBERSORT) algorithm. We identified differentially expressed genes among the dataset, then applied the weighted gene co-expression networks analysis (WGCNA) for network analysis. Furthermore, we combined Least Absolute Shrinkage and Selection Operator (LASSO) regression and random forest algorithm to identify characteristic genes involved in AS, then applied gene set variation analysis (GSVA) and gene set enrichment analysis (GSEA) to identify specific signaling pathways and potential molecular mechanisms underlying disease progression. In addition, we used the single cell database (GSE159677) to explore the correlation between expression of key genes and immune cell infiltration, then finally employed the connectivity map (CMAP) database to predict drugs which can target differentially expressed genes in AS. Collectively, our results provide a theoretical basis for further research on the etiology, pathophysiology, biological diagnostic markers, and therapeutic targets, of AS.

## 2. Materials and methods

### 2.1. Data sources

Search for “atherosclerosis” and “peripheral blood” in the National Center for Biotechnology Information GEO database, set the species to “Homo sapiens,” and exclude chip datasets with <6 samples. Finally, GSE20129 and GSE90074 were included. Download the Series Matrix File data file of GSE20129, the annotation file is GPL6104, and a total of 119 groups of patient expression profile data are included, of which 71 are normal and 48 are disease groups. Download the Series Matrix File data file of GSE90074 from the National Center for Biotechnology Information GEO public database. The annotation file is GPL6480, and a total of 143 groups of patient expression profile data are included, of which 50 are normal and 93 are disease groups. According to the GPL platform, the probe is converted to ID. When multiple probes correspond to the same gene ID, the average value is taken. Merge the 2 GEO datasets and use the Combat algorithm to correct the batch between the chips. The AS single-cell dataset GSE159677 is used for analysis, and a total of 6 groups of expression profile data are included, of which 3 are normal and 3 are disease groups.

### 2.2. Immunocyte infiltration analysis

CIBERSORT has been extensively used to analyze the type of immune cells in microenvironments. Using support vector regression, this method reduces the expression matrix of immune cell subtypes to its simplest form. In the present study, a total of 547 biomarkers are used to distinguish 22 human immune cell phenotypes, including T, B, plasma, and myeloid cells. The CIBERSORT algorithm was used to determine the relative proportion of 22 types of immune infiltrating cells in the downloaded datasets.

### 2.3. WGCNA analysis

By constructing a weighted gene co-expression network, we search for co-expressed gene modules, and explore the relationship between the gene network and T cells, as well as the core genes in the network. We use the WGCNA-R package to build a co-expression network of all genes in the dataset, and analyze the top 10,000 genes in terms of variance. The optimal soft-thresholding power is set to 4 to maximize the scale-free topology of the network. The weighted adjacency matrix is transformed into a topological overlap matrix, and a hierarchical clustering tree structure of the topological overlap matrix is constructed using hierarchical clustering methods. In addition, the minimum module size (minModuleSize) in the dynamic module identification stage is set to 50, meaning that each module contains at least 50 genes. Different branches of the clustering tree represent different gene modules, and different colors represent different modules. Based on the weighted correlation coefficient of the genes, the genes are classified according to their expression patterns, and genes with similar patterns are grouped into a module, thus all genes are divided into multiple modules through their gene expression patterns.

### 2.4. GO and KEGG pathway analysis

Firstly, we employed the Cluster Profiler to annotate genes in the module followed by functional correlation. Next, we performed Gene Ontology (GO) enrichment and Kyoto Encyclopedia of Genes and Genomes (KEGG) pathway analyses to evaluate related functional categories with the following thresholds for both analyses: *P* and q < .05.

### 2.5. Lasso regression and random forest identification of key genes

We use Lasso logistic regression and random forest algorithms for feature selection of disease diagnostic markers. In Lasso logistic regression, we model using the glmnet package, choose to use alpha = 1 for Lasso regression, and determine the optimal regularization parameter lambda through cross-validation (10-fold cross-validation). Through this method, we determine the most useful gene set for model prediction. In the random forest section, we use the randomForest package to build a forest containing 1000 decision trees. Each tree selects the square root number of features (i.e., mtry) without repetition when it is generated. We performed 50 times of feature importance perturbation (nPerm = 50) to evaluate the contribution of each feature to the model performance. The importance measure used is the increased mean square error (%IncMSE). Through this method, we can identify the most critical gene features for distinguishing disease status, further enhance our understanding of the disease mechanism, and assist in the discovery of diagnostic markers.

### 2.6. Analysis of single cell sequencing data

Firstly, we employed the Seurat package to process the data, then analyzed the location relationship between each cluster using the tSNE algorithm. Next, we employed the celldex package to annotate each cluster. Finally, we set logFC.threshold = 1 on FindAllMarkers to extract marker genes from the expression profiles of the individual cells. Each cell subtype was assigned unique marker genes based on the | avg log2FC | >1 and *P* val adj > .05 values.

### 2.7. GSEA analysis

We employed GSEA to sort out key genes based on the degree of differential expression between 2 types of samples using predefined gene sets. Next, we checked the sorted table to determine enrichment of the preset gene sets. We compared the signaling pathways regulated by the key genes (both up- and downregulated) using GSEA, then elucidated the underlying molecular mechanisms with the aim of explaining the difference in prognoses between the 2 groups. In this case, 1000 replacements were made, and the type of replacement was set to phenotype.

### 2.8. GSVA analysis

The transcriptome GSVA evaluates enrichment of transcriptome gene sets in a nonparametric, unsupervised manner. GSVA is first used to comprehensively score the gene set of interest, then determine the biological function of each sample by converting the gene level change into pathway level change. By downloading gene sets from the Molecular Signatures database, we used the GSVA algorithm to comprehensively score each gene set, and evaluated the potential biological functions of these gene sets.

### 2.9. Analysis of transcription regulation of key genes

In this study, we first employed the “RcisTarget” package in R to predict transcription factors. All calculations performed by RcisTarget are based on motif. There is a relationship between the total number of motifs in the database and the normalized enrichment score (NES) for each motif. Based on motif similarity and gene sequence, we inferred further annotation files based on the source data. To estimate the overexpression of each motif on the gene set, the area under the curve of each motif – motif pair must be calculated. The NES of each motif in our gene set was calculated based on its area under the curve distribution. A gene-motif ranking database was then built using rcistarget. hg19 motifdb. Cispont. 500bp is the index used in the Gene-motif ranking database.

### 2.10. Construction of miRNA network

MicroRNAs (miRNAs) regulate gene expression by either degrading or inhibiting mRNA translation. In the present study, we investigated whether some miRNAs in key genes regulate transcription or degradation of some dangerous genes. To this end, we obtained miRNAs associated with key genes through the Targetscan database and visualized the miRNA network via cytoscape software.

### 2.11. CMAP drug prediction

Broad Research Institute has developed a gene expression profile database called CMAP based on intervention gene expression. In this study, we sought to uncover the relationship between small molecule compounds, genes, and diseases. Briefly, 5 human cell lines were treated with 1309 small molecular drugs before and subjected to microarray analysis. Different drugs, at varying different concentrations were screened over time. We also predicted the targeted therapeutic drugs of diseases by analyzing differentially expressed genes of diseases.

### 2.12. Statistical analysis

R language (version 4.2.1) was used for all statistical analyses. A significance level of 0.05 was considered statistically significant.

## 3. Results

**Figure 1.**
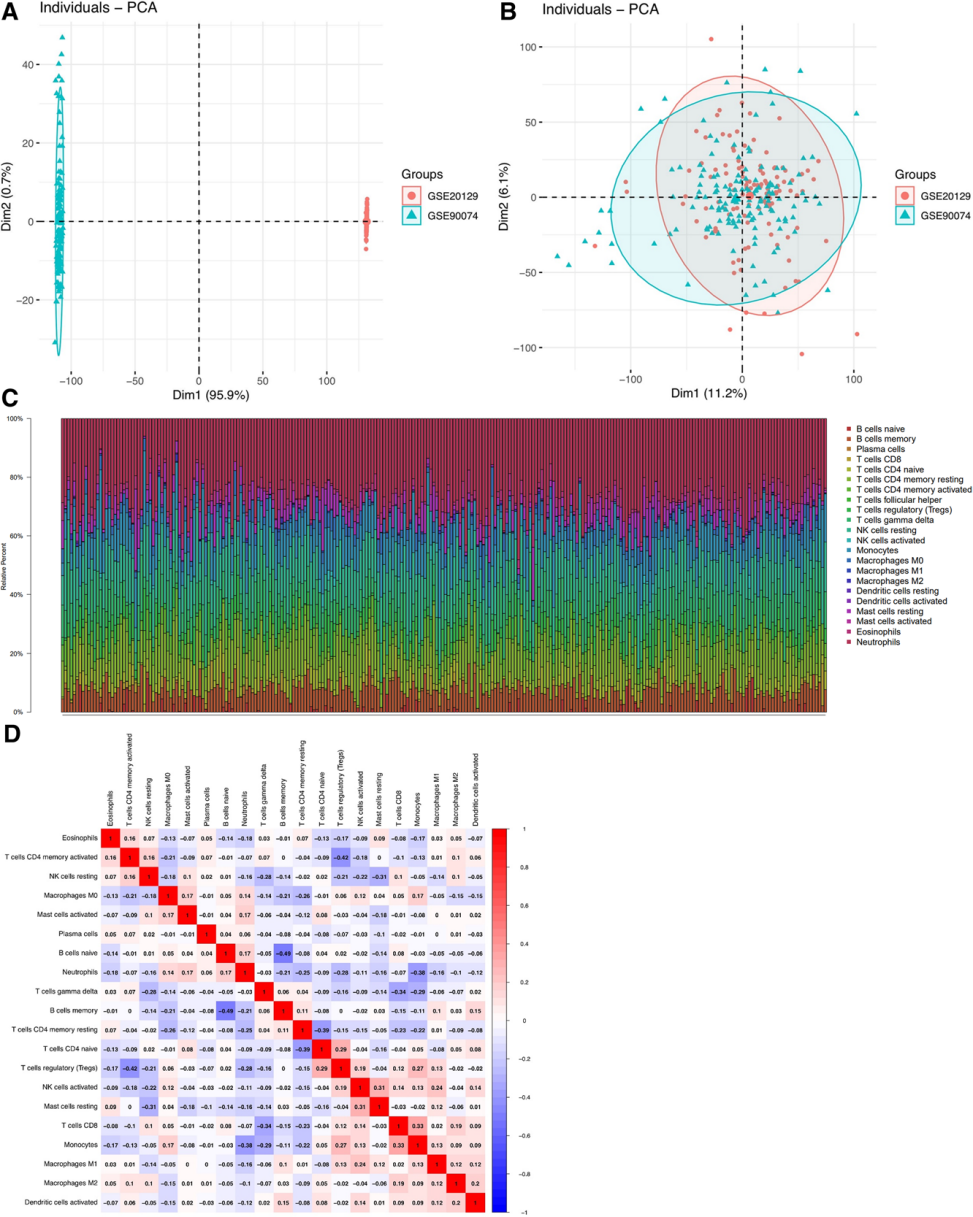
Immune infiltration profile of atherosclerotic tissues. (A and B) The data sets GSE20129 and GSE90074 were used in the combat algorithm to correct the batch effect before and after. (C) The relative percentage of 22 immune cell subsets in all samples. (D) Blue and red colors indicate positive correlation among 22 immune cells.

The GSE20129 and GSE90074 AS-related datasets were downloaded from the GEO database. The expression profile data comprised 262 patients, including 121 normal patients and 141 disease patients. To demonstrate differences between the before and after correction of the chip, the combat algorithm was applied whereas the PCA diagrams were used to correct the chip. The results showed that the inter-chip batch effect decreased after correction (Fig. 1A and B). In addition, the CIBERSORT algorithm was employed to calculate 22 immune cell scores based on the abundance of different subtypes of cells within these atherosclerotic samples. The immune cells of each patient as well as the correlation between their immune cells are shown in Figure 1C and D. Six subtype fractions of T cells were identified as characteristic data for WGCNA.

**Figure 2.**
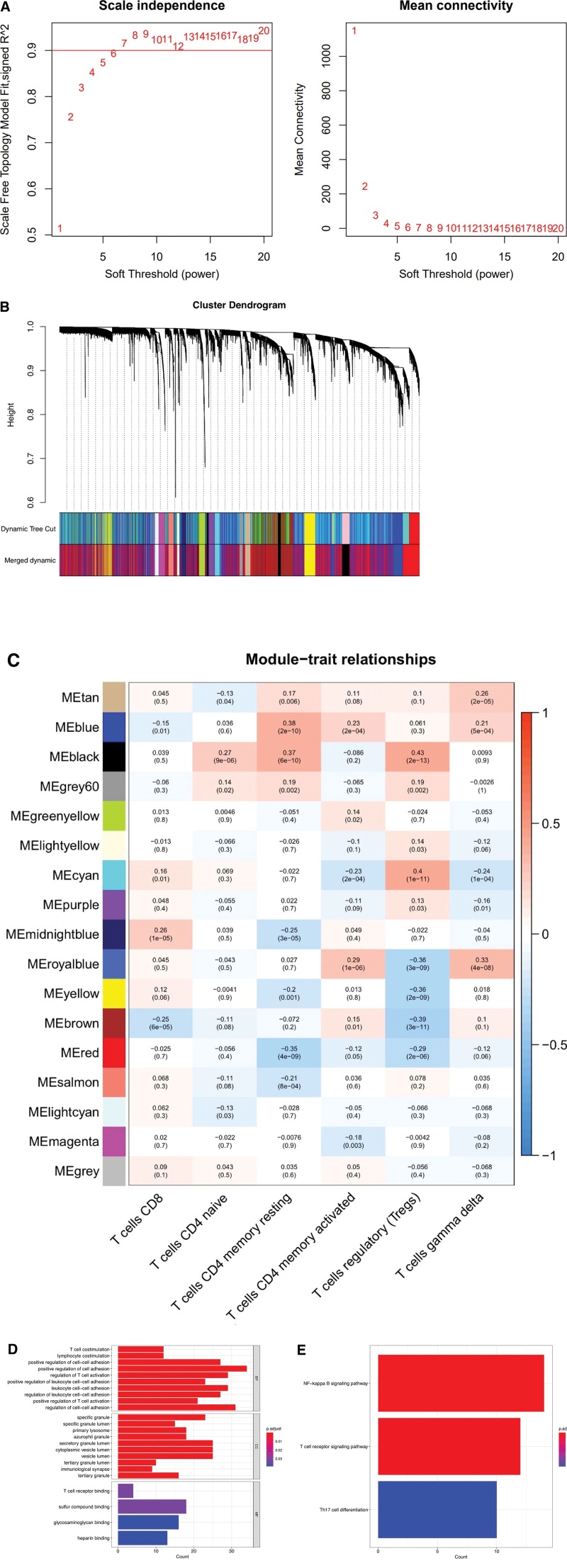
WGCNA analysis. (A) Scaleless index and mean connectivity of each soft threshold of atherosclerosis. (B) A dendrogram showing the atherosclerotic gene cluster, with different colors representing different modules. (C) A heat map displaying the correlations among the module genes and T cells, blue color indicates negative correlation, whereas red color indicates positive correlation. (D and E) GO-A histogram showing the KEGG enrichment results for the clusterprofiler. GO = Gene Ontology, KEGG = Kyoto Encyclopedia of Genes and Genomes, WGCNA = weighted gene co-expression networks analysis.

The WGCNA network was constructed based on the combined expression profiles to determine the co-expression network of the atherosclerotic cohort. Gene modules were detected based on the tom matrix. A total of 17 gene modules (Fig. 2A–C) were identified in this analysis, and the black module showed the greatest correlation with T cells (cor = 0.43, p = (2e−13)). To further explore the potential mechanism of underlying AS, we conducted GO and KEGG enrichment analysis on the black module using R package “cluster profile.” The results of GO enrichment analysis revealed that the main pathways were positive regulation of cell adhesion, regulation of T cell activation, positive regulation of Leukocyte cell–cell adhesion, etc (Fig. 2D). In the KEGG analysis, the main pathways were NF-κ B signaling pathway, T cell receiver signaling pathway, and Th17 cell differentiation signaling pathway (Fig. 2E).

**Figure 3.**
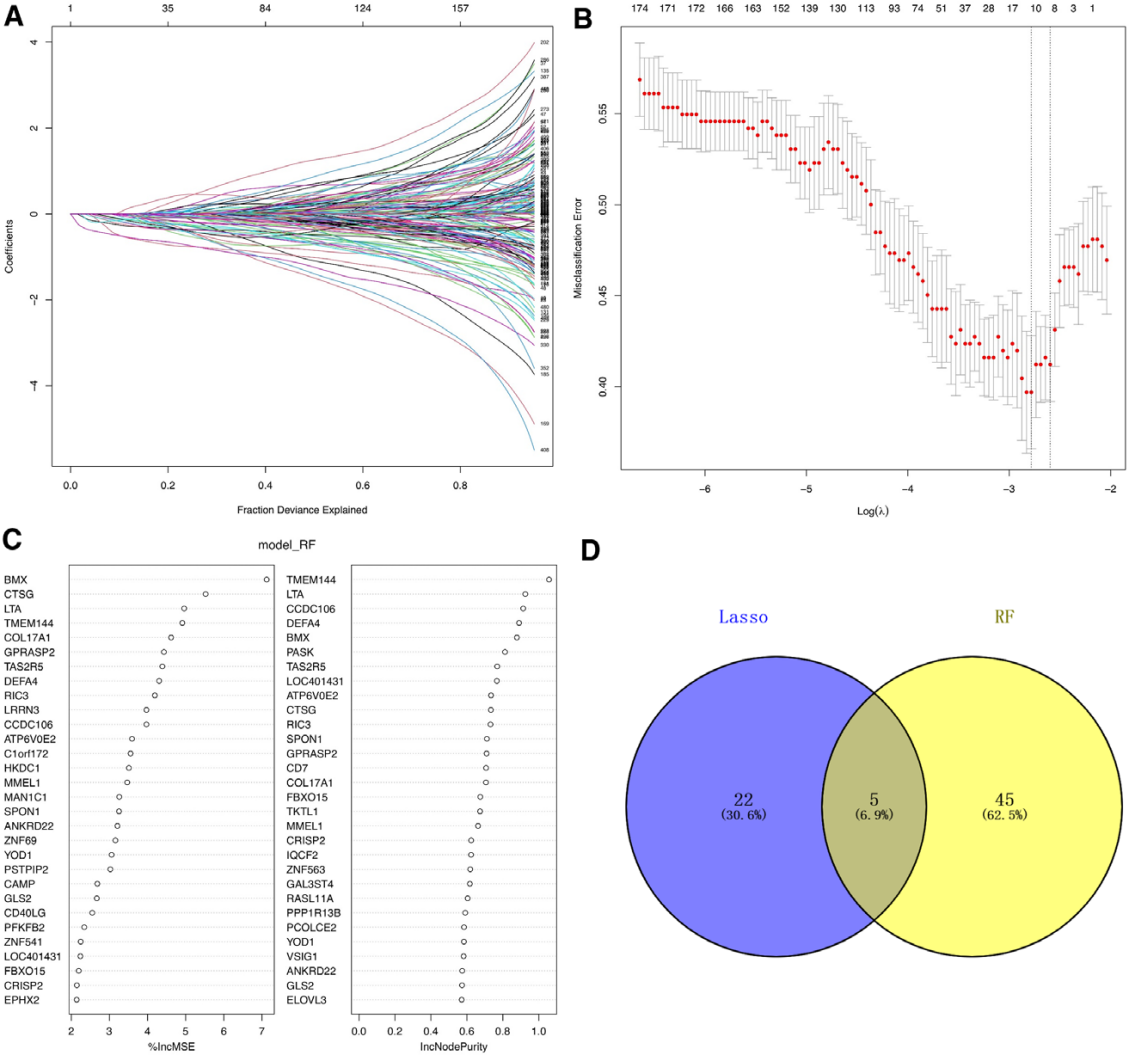
Identification of key genes of atherosclerosis by machine learning methods. (A) LASSO coefficient distribution of different genes. (B) Ten cross validation of tuning parameter selection in LASSO model. (C) The ranking of characteristic genes determined by the random forest algorithm. (D) Wayne diagram of the intersection genes between LASSO and random forest algorithm. LASSO = Least Absolute Shrinkage and Selection Operator.

The black module which had the strongest correlation with T cells in WGCNA was selected to identify the key genes involved in the occurrence of AS. To identify gene variants associated with AS, we used lasso regression and a random forest algorithm. We found that Lasso regression identified 27 genes as the characteristic genes of AS (Fig. 3A and B). In addition, we analyzed the characteristic genes involved in the pathogenesis of AS (Fig. 3C) using a random forest algorithm. The top 50 genes in the random forest were intersected with the characteristic genes identified using the Lasso regression algorithm, and 5 intersection genes (Fig. 3D) were retrieved. These 5 genes will be investigated in our follow-up research. They are: CCDC106, RASL11A, RIC3, SPON1, and TMEM144.

**Figure 4.**
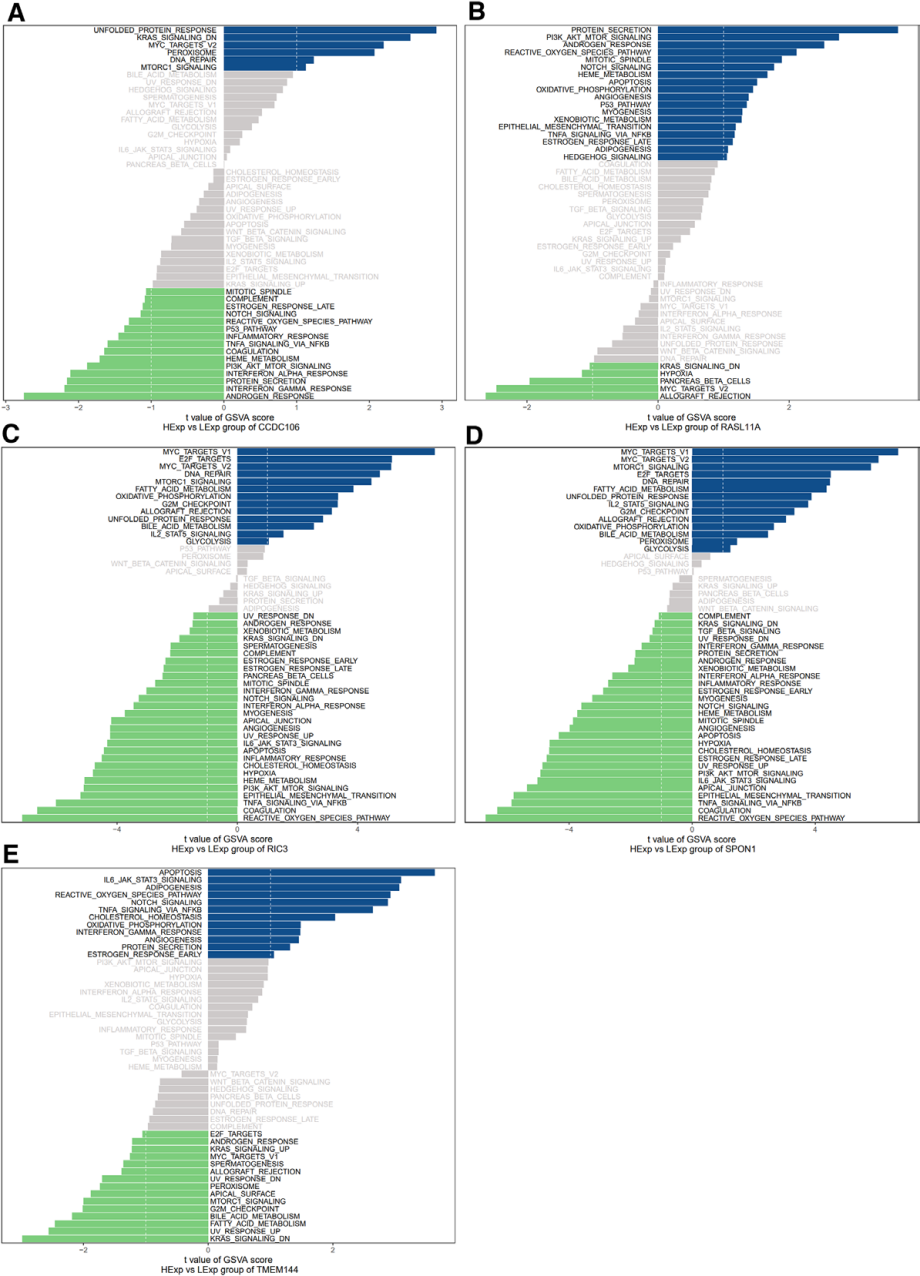
Pathway enrichment analysis based on GSVA. The vertical axis represents a change in signaling pathways in the corresponding samples with low or high key gene expression. The horizontal axis represents the degree of variation of each pathway in the gene set. The green bar displays the sample with high expression in the key gene, whereas the blue bar indicates opposite results. GSVA = gene set variation analysis.

To explore the potential molecular mechanisms by which the key genes regulated disease progression, we investigated the specific signal pathways involved by GSVA. The results showed that patients with high expression of CCDC106 were enriched with signal pathways such as UNFOLDED PROTEIN RESPONSE and KRAS SIGNALING DN (Fig. 4A); Patients with high expression of RASL11A were enriched with PROTEIN SECRETION, PI3K AKT MTOR SIGNALING, and other signal pathways (Fig. 4B). Patients with high expression of RIC3 are enriched with MYC TARGETS V1 and E2F TARGETS (Fig. 4C). Patients with high expression of SPON1 were enriched with MYC TARGETS V1 and MYC TARGETS V2 (Fig. 4D). Those with high expression of TMEM144 were enriched with APOPTOSIS, and IL6 JAK STAT3 SIGNALING pathways (Fig. 4E).

**Figure 5.**
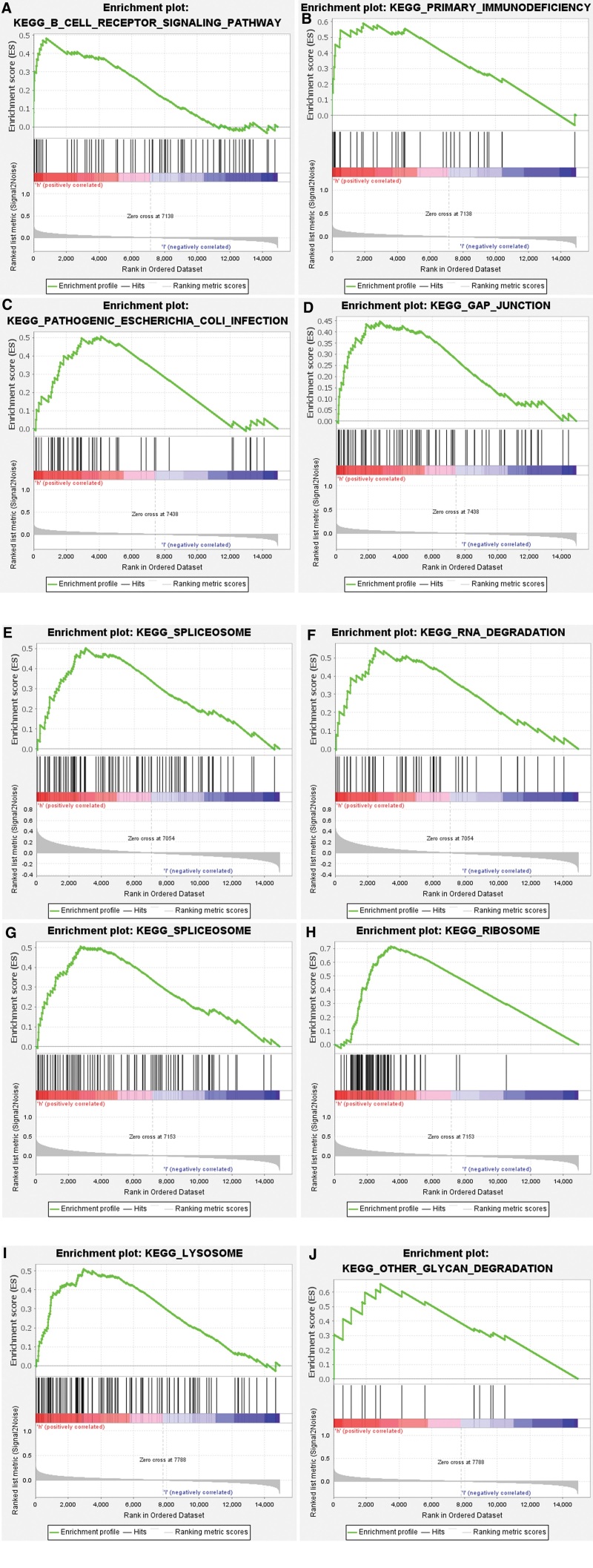
Enrichment analysis of the pathway based on GSEA. (A and B) CCDC106 was enriched in B cell receptor signaling pathway, primary immunodeficiency and other signal pathways; (C and D) RASL11A was enriched in pathogenic *Escherichia coli* infection, gap junction and other signal pathways; (E and F) RIC3 was enriched in SPLICEOSOME, RNA DEGRADATION, and other signal pathways; (G and H) SPON1 was enriched in the SPLICEOSOME, RIBOSOME, and other signal pathways; (I and J) TMEM144 enriched signal pathways such as LYSOOME and OTHER GLYCAN DEGRADATION. GSEA = gene set enrichment analysis.

In addition, GSEA results revealed that overexpression of CCDC106 was enriched in B CELL RECEPTOR SIGNALING PATHWAY, PRIMARY IMMUNODEFICIENCY, and other signal pathways(Fig. 5A and B); high expression of RASL11A was enriched in PATHOGENIC ESCHERICIA COLI INFECTION, GAP JUNCTION and other signal pathways(Fig. 5C and D); high expression of RIC3 was enriched in SPLICEOSOME, RNA DEGRADATION and other signal pathways (Fig. 5E and F); high expression of SPON1 was enriched in SPLICEOSOME, RIBOSOME, and other signal pathways(Fig. 5G and H); high expression of TMEM144 was enriched in signal pathways such as LYSOOME and OTHER GLYCAN DEGRADATION (Fig. 5I and J).

**Figure 6.**
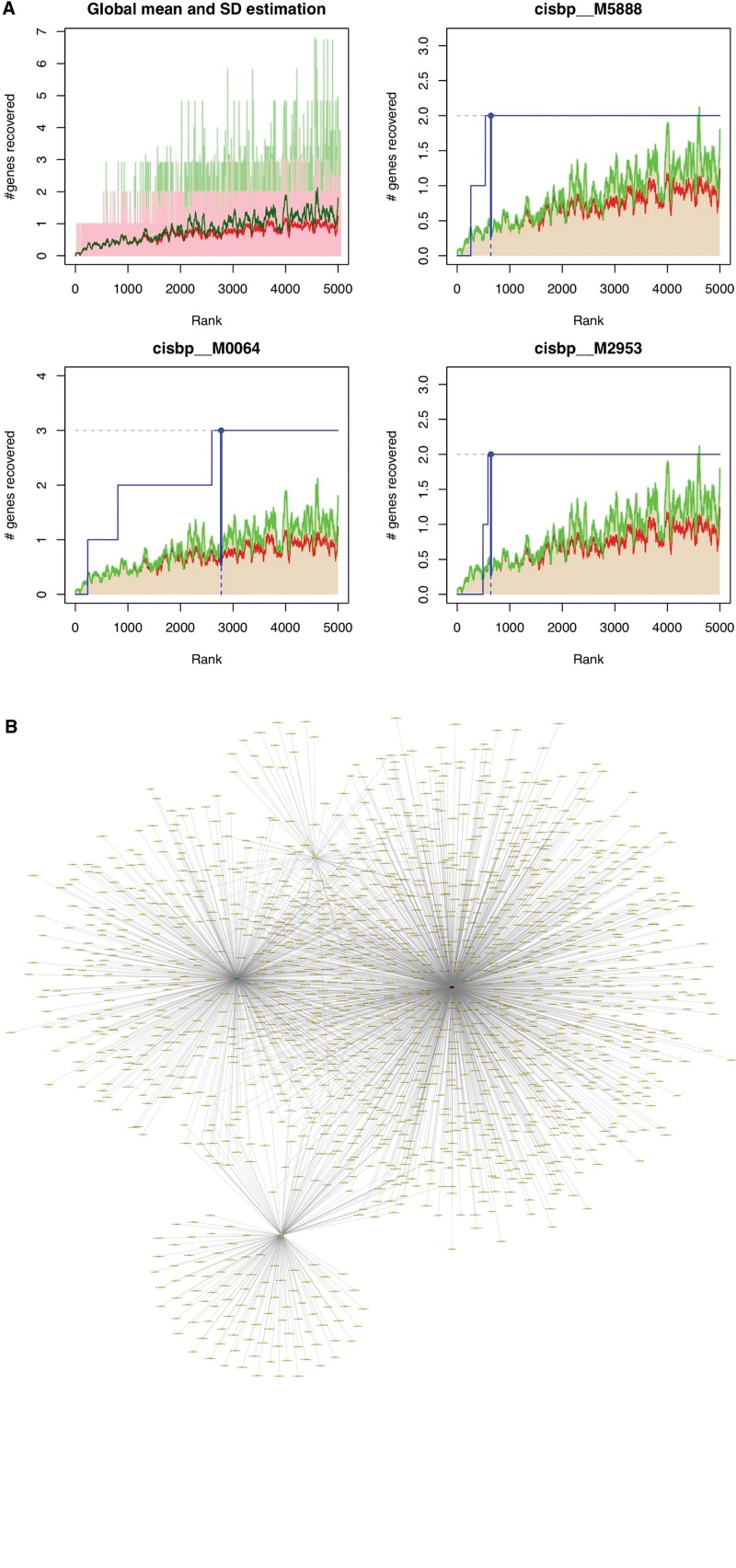
Motif transcriptional regulation analysis. (A) The 3 motifs with the highest AUC. The red line represents the average value of the recovery curve of each motif, the green line represents the average value + standard deviation, and the blue line represents the recovery curve of the current motif. The maximum distance point (mean + SD) between the current motif and the green curve was selected as the maximum enrichment level. (B) The miRNA network of key genes, purple represents mRNA, and yellow represents miRNA. miRNAs = microRNAs.

As part of this analysis, the 5 key genes were selected and found that they were regulated by multiple transcription factors and other mechanisms. The cumulative recovery curve was used to analyze these transcription factors (Fig. 6A). In addition, we used Motif-TF annotation and selected genes of importance. The analysis results demonstrated that the motif with the highest standardized enrichment score (NES: 8.38) was cisbp__M5888. Further, the targetscan database was used to reverse predict 5 key genes, including 1954 miRNAs and 1556 pairs of mRNA–miRNA relationship pairs, and the cytoscape was employed to visualize the results (Fig. 6B).

**Figure 7.**
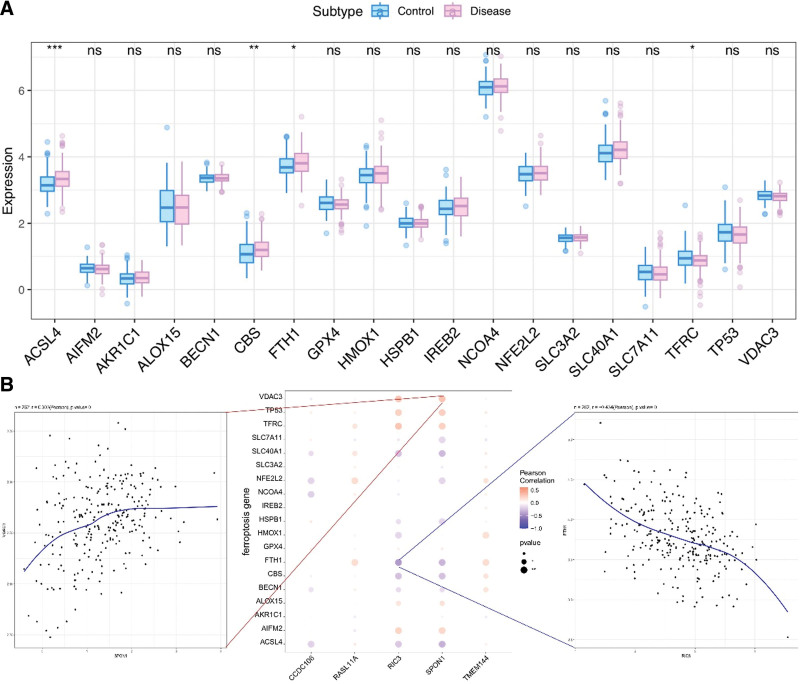
Correlation among atherosclerosis regulatory genes. (A) The difference in the expression of atherosclerotic disease regulatory genes, blue represents the control patients, and pink represents the disease patients. (B) The correlation analysis of atherosclerotic disease regulatory genes and key genes. Blue represents negative correlation and red represents positive correlation.

The GeneCards database (https://www.genecards.org/) was used to identify genes related to iron death. Differential expression of iron death-related genes between the groups. It was found that ACSL4, CBS, FTH1, and TFRC genes showed differential expression between the 2 groups of samples (Fig. 7A). A significant correlation was found between the expression levels of 5 genes and those of multiple diseases-related genes. RIC3 and FTH1 were significantly negatively correlated (r = ‐0.434), whereas SPON1 and VDAC3 were significantly positively correlated (*R* = 0.303) (Fig. 7B).

**Figure 8.**
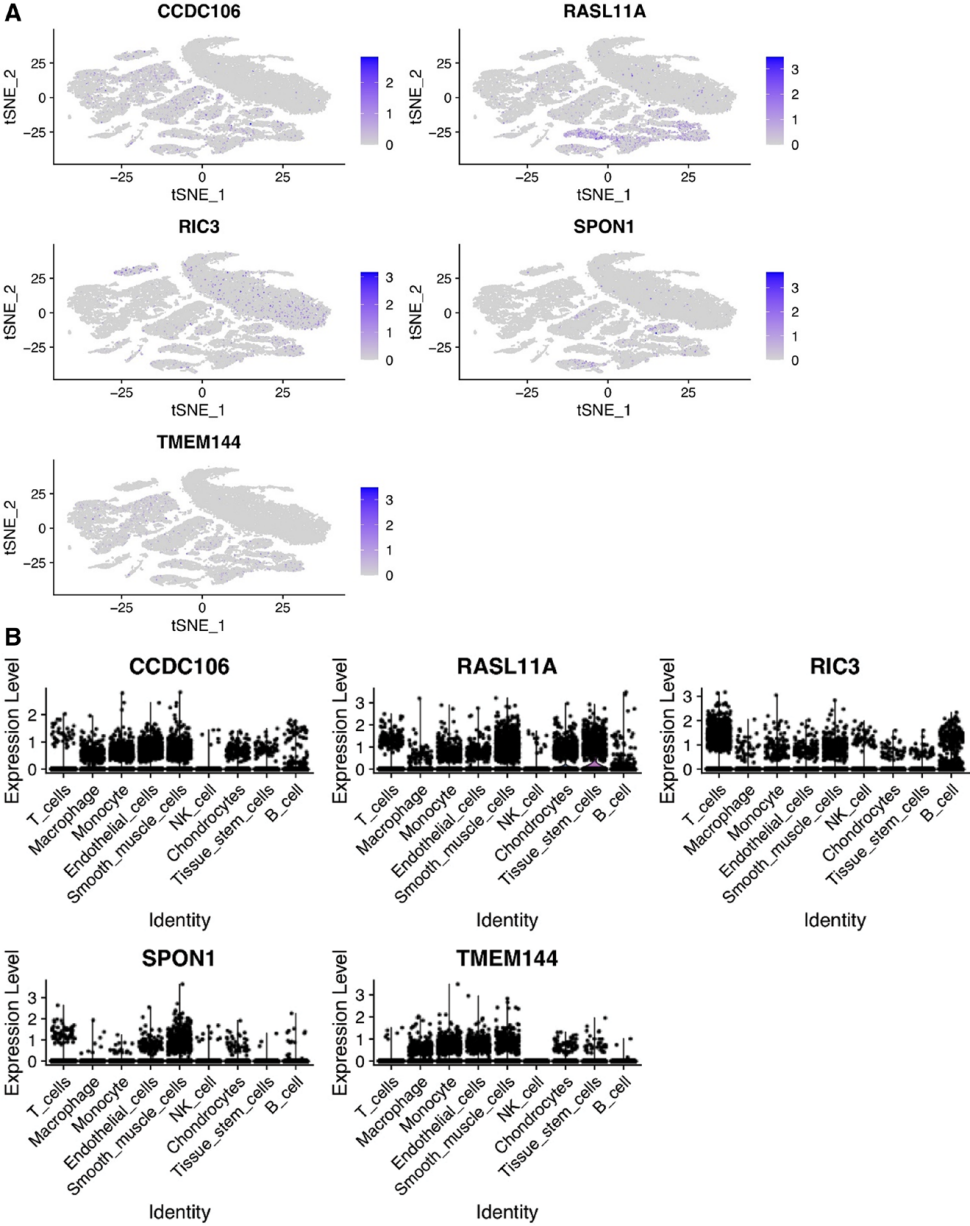
tSNE clustering of single-cell expression profiles.

The single cell data of GSE159677, cluster cells with tSNE algorithm, annotate each subtype with HumanPrimaryCellAtlas were downloaded as annotation data, and all subtypes were annotated with R packet SingleR_cell as Tissue_stem_cells, Chondrocytes, NK_cell, Smooth_muscle_cells, Endothelial_cells, Monocyte, Macrophage, and T_Cells. The expression of 5 key genes, CCDC106, RASL11A, RIC3, SPON1, and TMEM144, in 9 kinds of cells is shown in Figure 8A and B.

**Figure 9.**
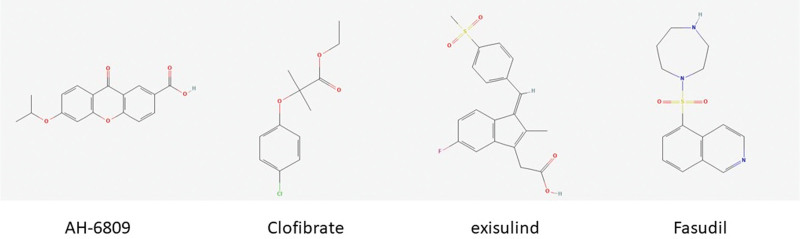
CMAP analysis identifies potential drugs based on the expression profile. CMAP = connectivity map.

The connectivity map database was used to predict drugs for each of the TOP150 genes differentially expressed in AS. The results showed that in AS, AH-6809, Clofibrate, exisulind, and Fasudil were the most significantly negatively correlated with the expression profile of disease disturbance, suggesting that drugs can alleviate or even reverse the disease state (Fig. 9).

## 4. Discussion

AS, a chronic inflammatory disease caused by lipid metabolism imbalance, vascular dysfunction and immune response, is a major cause of cardiovascular events.^[22]^ Previous estimates have shown that about 57.79 million people aged 30 to 70 in the world suffered from AS in 2020 alone, a 59.13% increase compared with 2000.^[3,23]^ According to the World Health Organization, the incidence and mortality rates of cardiovascular diseases are still high worldwide.^[24]^ Therefore, prospecting for biomarkers of AS coupled with predicting drug efficacies are imperative to effective management of AS.

In this study, we downloaded data for 121 and 141 non-AS arteries and AS artery samples, respectively, from GSE20129 and GSE90074 AS-related datasets from GEO database, then used WGCNA to screen the black gene module for functional enrichment which correlated most with T cells. Functional enrichment results revealed that the key genes were mainly involved in cell adhesion, T-cell-related and the NF-κ B signaling pathways. Studies have shown that inflammatory cells are recruited and infiltrated under the endothelium when adhesion molecules and chemokines are high, a phenomenon that results in plaque formation, rupture, and thrombosis.^[25–27]^ The NF-κ B signaling pathway is characterized by a classical pro-inflammatory signal system.^[28,29]^ Brand et al^[30]^ showed that AS activated the nuclear factor NF-κ B, which not only plays an important role in inflammatory response by stimulating cytokines, but also participates in production of various immune cells to regulate immune response. The resulting immune inflammatory reaction destroys the vascular wall, thereby affecting the function of vascular cells, and eliciting formation of atherosclerotic plaque.^[31]^

We used the Lasso regression and random forest algorithm to analyze 5 intersection genes, namely CCDC106, RASL11A, RIC3, SPON1, and TMEM144. The CCDC106 protein, was previously known as HSU79303, which contained a coiled coil domain.^[32]^ Members if the CCDC family promote tumor cell proliferation, while high levels of CCDC protein are associated with poor outcomes.^[33]^ Previous studies have shown that a functional CCDC106 gene can not only promote the degradation of TP53/p53 proteins but also inhibit their trading activities.^[34]^ TP53, a tumor suppressor, is involved in DNA damage and cell cycle regulation.^[35–38]^ P53 dysfunction was found to promote cancer development and progression. A previous study demonstrated that CCDC106 promotes cell proliferation both in vitro and in vivo by activating the AKT signaling pathway and upregulating expression of Cyclin A2 and Cyclin B1.^[39]^ However, the relationship between the CCDC106 gene and AS has not yet been clearly studied.

RASL11A, a new member of the Ras superfamily, is extremely conservative in eukaryotes. Functionally, they regulate a variety of cell processes, including cell growth and apoptosis.^[40,41]^ The Ras family of proteins serve as a fundamental signaling module and are involved in a wide range of physiological processes. They are small GTPases that alternate between GTP and GTP-binding states under the control of guanine nucleotide exchange factors and GTPase activators. In this way, Ras superfamily proteins function as molecular switches, regulating a numerous events across different cell compartments, and respond to many signals upstream.^[41–43]^ RASL11A and RASL11B are the 2 closest human homologs. A previous study^[39]^ demonstrated that phorbol ester upregulates RASL11B expression but downregulates that of RASL11A during maturation of THP-1 monocytes to macrophages.

AS is characterized by macrophages that produce TGF-β1, which stimulates smooth muscle cells to produce extracellular matrix molecules.^[44]^ Apart from transformation of monocytes into macrophages, response of the human coronary arterial smooth muscle cells to TGF plays a key role in the development of AS.^[44,45]^ Katrin et al^[39]^ observed the highest RASL11A and RASL11B expression levels in the opposite direction on human coronary artery smooth muscle cells, suggesting that RASL11A and RASL11B not only play opposing functions but also that their expression may be regulated by the same mechanism. To date, however, the actual mechanisms that regulate expression of RASL11B and RASL11A in coronary artery smooth muscle cells stimulated by TGF-β1 are poorly understood. Further research is needed to clarify how RASL11B and RASL11A expression is regulated during macrophage maturation.

RIC3 regulates folding, assembly and transportation of acetylcholine-gated ion channels are nicotinic acetylcholine receptor (nAChR).^[46]^ Acetylcholine-gated ion channels are nAChR. In non-excitatory cells, the cholinergic anti-inflammatory pathway is manifested through homologous-α7 nAChR. Apart from regulating cytokine release from macrophages,^[47–49]^ the cholinergic anti-inflammatory pathway also affects inflammation.^[50]^ Previous experimental results have revealed that RIC3 is expressed in immune cells, while its expression in mouse macrophage RAW264.7 cells is affected by immune activation.^[51]^ Moreover, Yael et al^[52,53]^ found a dynamic correlation between the induction of neuroinflammation in mice (EAE induction) and expression of RIC3. Particularly, RIC3 expression in human and mouse cells, lymphocytes and macrophages is affected by immune activation, indicating that they are necessary during inflammation. A large amount of clinical and experimental evidence shows that inflammation plays a key role in all stages of the AS process. Our research results once again prove that the pathogenesis of AS may have a certain correlation with inflammation.

SPON1 (F-spinin) belongs to thrombospondin, which contains thrombospondin type 1 repeat involved in matrix tissue and cell–cell interaction.^[54,55]^ HsamiR-188-5p and SPON1 play a role in angiogenesis.^[56,57]^ Some studies have shown that SPON1 is not only a sensitive plasma biomarker for myocardial infarction,^[58]^ but is also significantly correlated with adverse clinical outcomes in CHF patients. In vivo experimental data has shown that the SPON1 gene is not only involved in hypertension development but is also associated with heart failure and systolic dysfunction.^[59,60]^ In addition, SPON1 is associated with NT-proBNP, regulates macrophage activity and the response to damage-related molecular patterns/PAMPs. Stenemo^[59]^ and Dubin^[61]^ demonstrated that SPON1 is negatively correlated with left ventricular ejection fraction and GFR in HF patients. However, there has not been further analysis on the relationship between AS and SPON1 at present. Future research is needed to confirm the relationship between the 2 and explore the mechanism of SPON1 in the occurrence and development of AS. The relationship between TMEM144, a coding unit of transmembrane protein 144, with AS remains unclear. Future studies should determine whether TMEM144 can be used as a therapeutic target. In the present study, we used GSVA and GSEA to analyze the specific signal pathways involved in key genes, and screened out those associated with inflammation, immunity, etc. Accordingly, these genes may act through these markers and signal pathways to contribute to the development of AS. We can also study the mechanism of AS through this research direction.

AS is caused by iron deposition and lipid peroxidation in vascular endothelial cells.^[62,63]^ Studies on ferroptosis in cardiovascular diseases have shown that multiple signaling factors are either directly or indirectly involved in iron ptosis, thus affecting iron metabolism and lipid peroxidation.^[64]^ Iron ptosis has recently emerged as a therapeutic target in cardiovascular diseases.^[65]^ Results of the present study showed that genes associated with iron ptosis (ACSL4, CBS, FTH1, and TFRC) were differentially expressed between the diseased and control groups. Notably, RIC3 and FTH1 were significantly negatively correlated, whereas SPON1 and VDAC3 exhibited a significant positive correlation. These results set up a platform for future exploration of regulatory mechanisms underlying iron ptosis to guide development of new treatment strategies for AS.

Single cell sequencing technologies have made it possible for researchers to sequence a single cell from an organism.^[66,67]^ Technological advancement allows us to characterize molecular heterogeneity at the single-cell level thus bettering our understanding of the biological diversity within atherosclerotic plaques.^[68]^ We analyzed a single cell dataset (GSE159677) and identified key genes associated with immune, smooth muscle, endothelial and tissue stem cells in the present study. Recent studies have employed lineage tracking and single cell RNA sequencing to reveal the additional plasticity of SMC in AS. Our results were consistent with previous studies that have demonstrated that SMCs can generate cells similar to foam cells, macrophages, mesenchymal stem cells, and osteochondrocytes.^[69–71]^ Further explorations are needed to elucidate the mechanism underlying these phenotypes and the formation of atherosclerotic plaques.

Finally, we employed the Connectivity Map database to predict several related drugs, including AH-6809, Clofibrate, exisulind, and Fasudil. It is reported that there are many factors in the progression of AS, among which hyperlipidemia is one of the main factors. Clofibrate is a lipid-lowering drug used in clinical practice, which can improve the development of AS and has been proven to have a preventive effect on AS.^[72]^ Fasudil (HA-1077 or AT877), 5-(1,4-diazepan-1-ylsulfonyl) isoquinoline, is a nonselective ROCK inhibitor,^[73]^ which has shown good effects in the treatment of various cardiovascular diseases. It can maintain antioxidant capacity, weaken inflammation and apoptosis signals, and alleviate pathological angiogenesis. It has been proven to have vascular protective potential in experimentally induced severe unilateral limb ischemia models in mice, which provides important evidence for its possible involvement in the pathogenesis of AS.^[74]^ AH-6809 can inhibit the production of IL-1β and cAMP in macrophages amplified by PGE2,^[75]^ and the infiltration of monocytes and macrophages driven by inflammation within the vascular wall, as well as the formation of foam cells, are key stages in the development of AS.^[76]^ We speculate that AH-6809 may be involved in the occurrence and development of AS, but further research is needed to explore the correlation between AH-6809 and AS. Exisulind is an antitumor drug, and the relationship between exisulind and AS is currently unclear. In the future, further research is needed to confirm its effect on AS. Our results showed that these drugs can alleviate or even reverse the symptoms of disease expression profiles, as evidenced by the significant negative correlations with disease expression profiles. Clofibrate reportedly improves development of arteriosclerosis.^[77]^ Collectively, these results indicate that these drugs have potential to treat AS although animal experiments and clinical trials are needed to validate their efficacy and safety.

## 5. Conclusion

In summary, we used WGCNA to screen one module (black) with the highest correlation with T cells. We also used Lasso regression and random forest algorithm to identify 5 key genes (CCDC106, RASL11A, RIC3, SPON1, and TMEM144). Bioinformatic analyses revealed the relevant pathways regulated by these genes and the correlation between AS and inflammation and immune infiltration. Collectively, these results set up a platform for future exploration of the pathogenesis of AS and development of treatment therapies.

## Acknowledgments

We would like to thank all the research staff who made it possible to perform this study.

## Author contributions

**Data curation:** Meng Zhang, Guinan Yang.

**Investigation:** Xiaoqing Yuan.

**Methodology:** Xuebin Cui, Xiaoqing Yuan.

**Software:** Guinan Yang, Xuebin Cui.

**Validation:** Meng Zhang.

**Writing – original draft:** Xiaoxue Su, Liunianbo Du.

**Writing – review & editing:** Yuanmin Pei.
